# A Novel High-Throughput Method for Molecular Detection of Human Pathogenic Viruses Using a Nanofluidic Real-Time PCR System

**DOI:** 10.1371/journal.pone.0147832

**Published:** 2016-01-29

**Authors:** Coralie Coudray-Meunier, Audrey Fraisse, Sandra Martin-Latil, Sabine Delannoy, Patrick Fach, Sylvie Perelle

**Affiliations:** 1 Université Paris-Est, ANSES, Food Safety Laboratory, Enteric viruses Unit, 14 rue Pierre et Marie Curie, 94701 Maisons-Alfort Cedex, France; 2 Université Paris-Est, ANSES, Food Safety Laboratory, Identypath, 14 rue Pierre et Marie Curie, 94701 Maisons-Alfort Cedex, France; Hokkaido University, JAPAN

## Abstract

Human enteric viruses are recognized as the main causes of food- and waterborne diseases worldwide. Sensitive and quantitative detection of human enteric viruses is typically achieved through quantitative RT-PCR (RT-qPCR). A nanofluidic real-time PCR system was used to develop novel high-throughput methods for qualitative molecular detection (RT-qPCR array) and quantification of human pathogenic viruses by digital RT-PCR (RT-dPCR). The performance of high-throughput PCR methods was investigated for detecting 19 human pathogenic viruses and two main process controls used in food virology. The conventional real-time PCR system was compared to the RT-dPCR and RT-qPCR array. Based on the number of genome copies calculated by spectrophotometry, sensitivity was found to be slightly better with RT-qPCR than with RT-dPCR for 14 viruses by a factor range of from 0.3 to 1.6 log_10_. Conversely, sensitivity was better with RT-dPCR than with RT-qPCR for seven viruses by a factor range of from 0.10 to 1.40 log_10_. Interestingly, the number of genome copies determined by RT-dPCR was always from 1 to 2 log_10_ lower than the expected copy number calculated by RT-qPCR standard curve. The sensitivity of the RT-qPCR and RT-qPCR array assays was found to be similar for two viruses, and better with RT-qPCR than with RT-qPCR array for eighteen viruses by a factor range of from 0.7 to 3.0 log_10_. Conversely, sensitivity was only 0.30 log_10_ better with the RT-qPCR array than with conventional RT-qPCR assays for norovirus GIV detection. Finally, the RT-qPCR array and RT-dPCR assays were successfully used together to screen clinical samples and quantify pathogenic viruses. Additionally, this method made it possible to identify co-infection in clinical samples. In conclusion, given the rapidity and potential for large numbers of viral targets, this nanofluidic RT-qPCR assay should have a major impact on human pathogenic virus surveillance and outbreak investigations and is likely to be of benefit to public health.

## Introduction

Human enteric viruses constitute a serious public health concern, since they are capable of causing a variety of acute illnesses, including the most commonly reported acute gastrointestinal illness. They are mainly transmitted *via* the fecal-oral route either by person-to-person contact or by ingestion of contaminated water and food, particularly shellfish, soft fruits and vegetables. Enteric viruses are shed in enormous quantities in feces (10^9^ to 10^10^/g) and have an infectious dose on the order of tens to hundreds of virions. Enteric viruses are host-specific and are not capable of replicating in the environment, but they survive for long periods of time on food or food contact surfaces or in water (ground, surface, and drinking water) [1]. These characteristics enable enteric viruses to play a significant role in food- and waterborne outbreaks. Aside from noroviruses, which have been recognized as the largest cause of outbreaks, the viruses most often implicated in outbreaks include hepatitis viruses (hepatitis A virus and hepatitis E virus), rotavirus, adenovirus (40, 41), astrovirus, enterovirus [2, 3, 4, 5, 6, 7]. Additional viruses of lesser epidemiologic importance include human bocavirus, cosavirus, parvovirus, sapovirus, tick-borne encephalitis virus (TBEV), Aichi virus, and coronavirus [8, 9, 10, 11].

Tools for rapid detection of viral pathogens are important for analyzing clinical, environmental and food samples. Detection of these enteric viruses based on their infectivity is complicated by the absence of a reliable cell culture method and the low levels of contamination of food and environmental samples [12,13]. To date, real time RT-PCR has been one of the most promising detection methods due to its sensitivity, specificity, and speed. Recently, the ISO/TS 15216–1 and 15216–2 standards covering real time RT-PCR for both quantitative determination and qualitative detection of NoV and HAV in foodstuffs were published [14, 15, 16].

The aim of this study was to develop real time RT-PCR assays for detection of a total of 19 human enteric viruses (including 3 genogroupes of norovirus and 4 coronaviruses) and two control process viruses (mengovirus and murine norovirus) generally used for monitoring the recovery of viral foodstuff extraction methods. Limits of detection of the viral genomes were determined with the conventional RT-qPCR system and with the Fluidigm’s BioMark System by using the qualitative nanofluidic real-time RT-PCR array and the quantitative digital RT-PCR array. The advantages of these new detection techniques were determined by detecting and quantifying pathogenic viruses in clinical samples.

## Methods

### Viruses and cells

HAV strain HM175/18f, clone B (VR-1402), was obtained from the American Type Culture Collection (ATCC). This clone replicates rapidly and has cytopathic effects in cell culture [17]. HAV stock was produced by propagation in foetal rhesus monkey kidney (FRhK-4) cells (ATCC, CRL-1688) [18] and titrated by plaque assay [19]. The titer of viral production was established in HAV RNA genomic copies with an RT-qPCR standard curve obtained with the ten-fold diluted *in vitro* RNA transcripts. Based on this approach, HAV stocks had titres of 9.33 x 10^8^ genome copies / mL.

Dr. H. Virgin from Washington University in the USA provided ANSES’s Fougères Laboratory in France with MNV-1 (CW1 strain) which was then propagated in mouse leukemic monocyte macrophage (RAW 264.7, ATCC TIB-71) cell line [20]. MNV-1 stock was produced as previously described [21]. The extracted RNA was confirmed with RT-qPCR and quantified by measuring absorbance at 260 / 280 nm with a NanoDrop ND-1000. Based on this approach, the production stock of MNV-1 had titres of approximately 1.36 x 10^12^ genome copies / mL.

Mengovirus (strain MC_0_) was obtained from clarified supernatant provided by Dr. Albert Bosch from the “Enteric Virus Group” of the University of Barcelona. Mengovirus stock was produced by propagation in HeLa cells (ATCC, CCL-2^™^) [22]. The extracted RNA was confirmed with RT-qPCR and quantified by measuring absorbance at 260 / 280 nm with a NanoDrop ND-1000. Based on this approach, the production stock of MNV-1 had titres of approximately 6.68 x 10^11^ genome copies / mL.

Rotavirus strain Wa (human rotavirus) was obtained from the Pasteur Institute (Paris, France) and was propagated in MA-104 rhesus monkey epithelial cell line (ATCC CRL-2378) [23]. The extracted RNA was confirmed with RT-qPCR and quantified by measuring absorbance at 260 / 280 nm with a NanoDrop ND-1000. Based on this approach, the production stock of Wa had titres of approximately 3.21 x 10^11^ genome copies / mL.

Four viral strains were obtained from infected cell culture supernatants: two enterovirus strains (human B6 coxsackievirus (Schmitt strain: ATCC^®^ VR-1037AS/MK^™^) and human echovirus 19 (Burke strain)), one Adenovirus 40 strain (ATCC VR-931) and one Astrovirus GI strain.

### Viruses and stools

The study was conducted in accordance with the ethics principles of the Declaration of Helsinki. Hepatitis A virus infection is a notifiable disease in France. The current system of mandatory reporting was approved by the Commission Nationale de l’Informatique et des Libertés (deliberation n° 02–082, November 19 2002). Patients receive oral and written information on the finality of the notification and on the modalities of information recording. This information is available on line on the web site of the Institut de Veille Sanitaire (IVS) at http://www.invs.sante.fr/content/download/6498/42945/version/2/file/fiche_info_patient.pdf for HAV samples and on the web site of the NRC at www.cnr-ve.org for enteric virus samples. All clinical and biological parameters are treated anonymously. The virological surveillance of strain diversity is performed on stored samples obtained for hepatitis A diagnosis (no need for any additional blood draw). Diagnostic laboratories are asked to contribute to HAV and enteric virus strains surveillance by sending samples to the National Reference Centre (NRC). The study was not specifically approved by an ethics committee. Human samples were collected before the study and they are anonymously collected and analyzed.

The following human stool samples were provided by the National Reference Center (NRC) for Enteric Viruses (Dijon, France): adenovirus 41 (stool n°E5669), astrovirus GI (E4883), norovirus GI (E5486; E5569; E8050), norovirus GII (E6929; E6618; E7859; E7022; E6992), rotavirus (RV G12P8 = E7622; RV G1P8 = E8097), Aichi virus (E6841) and norovirus GII.13 + norovirus GIV.

The following human stool samples were provided by the National Reference Center (NRC) for HAV/HEV (Villejuif, France): HAV GIA (stools no. 780627147; 1181216151), HAV GIB (1280210015; 1280514230), HEV G3c (1280511146), HEV G3f (1280418084; 1280530128) and HEV G4 (1280615097; 1280522166).

The faecal samples were suspended in 1X Phosphate Buffered Saline (PBS), pH 7, to obtain a final 10% suspension (w/v), vortexed and centrifuged at 3000 g for 30 min at 4°C. Aliquots of 100 μL supernatant were kept frozen at -80°C for later use.

The extracted genomic RNA/DNA of adenovirus, astrovirus and rotavirus were confirmed with RT-qPCR and quantified by measuring absorbance at 260 / 280 nm with a NanoDrop ND-1000.

The extracted genomic RNA of norovirus GI, GII and GIV, sapovirus, Aichi virus, HAV and HEV were confirmed and quantified with RT-qPCR by using *in vitro* RNA standard curves (see “DNA and RNA standards”).

### DNA and RNA standards

Sequences from reference strains were inserted into recombinant plasmids (Table 1). The HEV, HAV, NoV GI and NoV GII cDNA were cloned in pGEM-T Easy vector (Promega, Charbonnières-les-Bains, France) and propagated in *E*. *coli* One Shot^®^ TOP10F’ (Life technologies, Saint Aubin, France). High-quality DNA plasmids containing HAV or NoV regions were purified using the Qiagen Plasmid midi kit (Qiagen, Courtaboeuf, France) according to the manufacturer’s protocol. Then, NoV GI plasmid was digested with NCOI (Life technologies), and HEV DNA, HAV DNA and NoV GII DNA plasmids were digested with SpeI (Life technologies) and transcripts were obtained by using a MEGAscript^®^ kit (Life technologies) according to the manufacturer’s protocol. Synthesized RNA were treated with Turbo^™^ DNase (Life technologies) according to the manufacturer’s protocol in order to remove the DNA template following transcription, and purified by using the MEGAclear^™^ kit (Life technologies). The synthesized DNA and RNA were confirmed with (RT)-qPCR and quantified by measuring absorbance at 260 / 280 nm with a NanoDrop ND-1000 (Thermoscientific, Courtaboeuf, France) and the free software available on the “http://endmemo.com/bio/dnacopynum.php” website. Aliquots of 10μL containing 10^9^ genome copies / μL were kept frozen at -20°C for later use and used as standards.

**Table 1:** GenBank accession number for viral sequences used to obtain recombinant plasmids.

Virus	Reference sequence	Position of the genomic sequence cloned	Plasmid used
Hepatitis A virus	M59808.1	39–518	pGEM-T Easy vector
Hepatitis E virus	AB097812	5301–5371	pGEM-T Easy vector
Norovirus GI	M87661	5257–5413	pGEM-T Easy vector
Norovirus GII	X86557	4981–5135	pGEM-T Easy vector
Norovirus GIV	JQ613567	4961–5140	pBluescriptIISK
Sapovirus	NC_006269	5051–5200	pBluescriptIISK
Aichi virus	AB040749	241–350	pBluescriptIISK
TBEV	U27495	11031–11141	pBluescriptIISK
Parvovirus B19	AB550331	2221–2420	pBluescriptIISK
Cosavirus	NC_012800	701–860	pBluescriptIISK
Bocavirus	NC_012042	2511–2700	pBluescriptIISK
229E	AF304460	25351–25530	pBluescriptIISK
HKU1	HM034837.1	28751–28940	pBluescriptIISK
NL63	JX504050	26191–26380	pBluescriptIISK
OC43	JN129835.1	28791–28940	pBluescriptIISK

Sapovirus, TBEV, norovirus GIV, Aichi virus, 229E, HKU1, cosavirus, OC43 and NL63 cDNA were cloned into the pBluescriptIISK+ vector by Genecust (Dudelange, Luxembourg). All recombinant plasmids were purified by Genecust and used to produce RNA transcripts. Sapovirus, TBEV, norovirus GIV, 229E, cosavirus and NL63 DNA plasmids (0.5 μg) were digested with *EcoR*V (Life technologies) and Aichi virus, HKU1 and OC43 DNA plasmids were digested with *Spe*I (Life technologies).

Digested plasmids were transcribed by using the MEGAscript^®^ kit (Life technologies) according to the manufacturer’s protocol. Synthesized RNA was treated with Turbo^™^ DNase (Life technologies) according to the manufacturer’s protocol in order to remove the DNA template following transcription, and purified by using the MEGAclear kit (Life technologies) according to manufacturer’s instructions. The synthesized RNA was confirmed with RT-qPCR and quantified by measuring absorbance at 260 / 280 nm with a Nanodrop ND-100 (Thermoscientific, France) and the free software available on the “http://endmemo.com/bio/dnacopynum.php” website. RNA stocks were diluted to contain 10^9^ copies / μL. Aliquots of 10 μL were kept frozen at -20°C for later use as standards.

Bocavirus and parvovirus cDNA were cloned into the pBluescriptIISK+ vector by Genecust (Dudelange, Luxembourg). Both recombinant plasmids were purified by Genecust. DNA plasmids (0.5 μg) were digested with *Spe*I (Life technologies) to be linearized. The synthesized DNA was confirmed with qPCR and quantified by measuring absorbance at 260 / 280 nm with a Nanodrop ND-100 (Thermoscientific, France) and the free software available on the “http://endmemo.com/bio/dnacopynum.php” website. DNA stocks were diluted to contain 10^9^ copies / μL. Aliquots of 10 μL were kept frozen at -20°C for later use as standards.

### Nucleic acid extraction

Adenovirus 40, adenovirus 41, astrovirus, rotavirus, coxsackievirus B6, MNV-1, mengovirus, HAV (HM175/18f, HAV GIA and HAV GIB), HEV (HEV G3c, HEV G3G et HEV G4), NoV (GI, GII, GII+GIV), sapovirus, echovirus 19, Aichi virus, and astrovirus DNA or RNA were extracted using the NucliSens^®^ easyMAG^™^ platform (Biomérieux Marcy l’Etoile, France) for total nucleic acid extraction by the ‘off board Specific A” protocol according to manufacturer’s instructions. Nucleic acids were eluted in 100 μL of elution buffer and stored at -80°C.

Adenovirus 41, astrovirus, rotavirus, coxsackievirus B6, MNV-1 and mengovirus DNA or RNA were confirmed with (RT)-qPCR and quantified by measuring absorbance at 260 / 280 nm with a NanoDrop ND-1000 (Thermoscientific, Courtaboeuf, France) and used as standards.

Extracted RNA from HAV (HM175/18f, HAV GIA and HAV GIB), HEV (HEV G3c, HEV G3G and HEV G4), NoV (GI, GII, GII+GIV), sapovirus, echovirus 19, Aichi virus, adenovirus 40 and astrovirus were quantified with (RT)-qPCR using standards (*in vitro* transcripted RNA, plasmidic DNA, extracted genomic RNA).

### Primers and probes

The primers and probes used to detect all the viruses of this study are described in Table 2. Those used to detect NoV GI, NoV GII, HAV and mengovirus are described in ISO/ TS 15216–1 / 15216–2 (2013). All the primers and probes were purchased from Life Technologies or Eurofins MWG Operon (Les Ulis, France).

**Table 2:** Primers and probes used in this study. (F: Forward; R: Reverse; P: Probe)

Virus	Specificity	Sequence (5’—3’)	Location Size	Target	Reference
					(+/-*adapted for this study*)
Hepatitis A virus	All genotypes	F—TCA CCG CCG TTT GCC TAG	68–85	5’UTR	[22]
		R—GGA GAG CCC TGG AAG AAA G	241–223	M14707	[22]
		P—FAM-CCT GAA CCT GCA GGA ATT AA-MGB	169–150	174bp	[22]
Hepatitis E virus	All genotypes	F—CGG TGG TTT CTG GGG TGA C	5260–5278	ORF2/ORF3	[35]
		R—AGG GGT TGG TTG GAT GAA TAT AG	5330–5308	M73218	[35]
		P—FAM-GGG TTG ATT CTC AGC CCT TCG C-BHQ1	5280–5301	71bp	[35]
Rotavirus	Serotype A	F—ACC ATC TWC ACR TRA CCC TC	963–982	NSP3 (segment 7)	[36], adapted
		R—GGT CAC ATA ACG CCC C	1049–1034	X81436	[37]
		P—FAM-ACA ATA GTT AAA AGC TAA CAC TGT CAA-BHQ1	990–1016	87pb	[36], adapted
Norovirus	Genogroup I	F—CGC TGG ATG CGN TTC CAT	5291–5308	5' end of ORF2	[38]
		R—CCT TAG ACG CCA TCA TCA TTT AC	5376–5334	M87661	[39]
		P—FAM-TGG ACA GGA GAY CGC RAT CT-BHQ1	5321–5340	86bp	[39]
Norovirus	Genogroup II	F—ATG TTC AGR TGG ATG AGR TTC TCW GA	5012–5037	5' end of ORF2	[40]
		R—TCG ACG CCA TCT TCA TTC ACA	5080–5100	X86557	[41]
		P—FAM-AGC ACG TGG GAG GGC GAT CG-BHQ1	5042–5061	89bp	[40]
Norovirus	Genogroup IV	F—GGA TGC GRT TCT CAG ACT	4986–5003	ORF1-ORF2	This study
		R—TCT TCA TTC ACA AAR TCG GGA G	5055–5034	JQ613567	This study
		P—FAM-TGG GAG GGG GAT CGC GAT CT-BHQ1	5012–5031	70bp	[42]
Sapovirus	GG 1, 2, 4, 5	F—GAC CAG GCT CTC GCY ACC TAC	5074–5094	Polymerase / capsid junction	[43]
		R—CCC TCC ATY TCA AAC ACT AWT TTG	5177–5154	NC_006269	[43]
		P—FAM-CCC ACT GGG TCA RGT ACT GTA C-BHQ1	5135–5114	104bp	This study
Aichi virus	/	F—CCA GCC TGA CGT ATC ACA GG	268–287	5’ UTR	[44]
		R—CAC AAT TGC CAC GTG AGC AGC TT	329–307	AB040749	[44]
		P—FAM-CTG TGT GAA GYC C-MGB	288–300	62bp	[44]
Astrovirus	/	F—TCT YAT AGA CCG YAT TAT TGG	2209–2229	ORF 1a	[45]
		R—TCA AAT TCT ACA TCA TCA CCA A	2322–2301	NC_001943	[45]
		P—FAM-CCC CAD CCA TCA TCA TCT TCA TCA-BHQ1	2295–2272	114bp	[45]
Adenovirus	40 and 41 (serotype F)	F—CTC GAC ATG ACT TTT GAG GT	20256–20275	Hexon protein	[45], adapted
		R—GTA GAC GGC CTC GAT GAC	20375–20358	NC_001454	[45], adapted
		P—FAM-AGG ATG AGC CCA CAC TTC TYA TGB-BHQ1	20290–20302	120bp	[45], adapted
Coronavirus (human)	229 (α-coronaV)	F—CAT ACT ATC AAC CCA TTC AAC AAG	25374–25397	glycoprotein	[46]
		R—CAC GGC AAC TGT CAT GTA TT	25510–25491	AF304460	[46]
		P—FAM-ATG AAC CTG AAC ACC TGA AGC CAA TCT ATG-BHQ1	25480–25451	137bp	[47]
	HKU1 (β-coronaV)	F—TCC TAC TAY TCA AGA AGC TAT CC	28775–28797	phosphoprotein	[46]
		R—AAT GAA CGA TTA TTG GGT CCA C	28921–28900	HM034837.1	[46]
		P—FAM-TYC GCC TGG TAC GAT TTT GCC TCA-BHQ1	28808–28831	147bp	[47]
	NL63 (α-coronaV)	F—GTT CTG ATA AGG CAC CAT ATA GG	26215–26237	phosphoprotein	[46]
		R—TTT AGG AGG CAA ATC AAC ACG	26357–26337	JX504050	[46]
		P—FAM-CGC ATA CGC CAA CGC TCT TGA ACA-BHQ1	26326–26303	143bp	[47]
	OC43 (β-coronaV)	F—CAT ACY CTG ACG GTC ACA ATA ATA	28812–28835	glycoprotein	[46]
		R—ACC TTA GCA ACA GTC ATA TAA GC	28921–28899	JN129835.1	[46]
		P—FAM-TGC CAA AGA ATA GCC ART ACC TAG T-BHQ1	28889–28865	110bp	[47], adapted
Tick-born-encephalitis virus	European and Far-Eastern subtypes	F—GGG CGG TTC TTG TTC TCC	11054–11071	3’NCR	[48], adapted
		R—ACW CAT CAC CTC CTT GTC AGA CT	11121–11099	U27495	[48], adapted
		P—FAM-TGA GCC ACC ATC ACC CAG ACA CA-BHQ1	11073–11095	68bp	[48], adapted
Parvovirus	B19	F—CCC CGG GAC CAG TTC AGG	2241–2258	NS	[49]
		R—CCC CTY ACA CCR TCC CAC AC	2393–2374	AB550331	[49]
		P—FAM-ATC ATY TGT CGG AAG CYC AGT TTC CTC CG-BHQ1	2262–2290	153bp	[49]
Enterovirus	/	F—GCC CCT GAA TGC GGC	334–348	Polyprotein	[50]
		R—GAT TGT CAC CAT AAG CAG C	481–464	AJ295199	[51], adapted
		P—FAM-CGG AAC CGA CTA CTT TGG GTG TCC GT-BHQ1	416–441	148pb	[51]
Cosavirus	/	F—TTG TAG YGA TGC TGT RTG TGT GTG	735–758	5’-UTR	[52], adapted
		R—CCA YTG TGT GGG TCC TTT CG	827–808	NC_012800	[52], adapted
		P—FAM-CYC ACA GGC CRR AAG CCC TGT C-BHQ1	783–807	93bp	[52], adapted
Bocavirus	hBoV2	F—TCA GAC CAA GCG ACG AAG AC	2531–2550	Gene *NP-1*	[53]
		R—CTC TAG CAA GYC TAG TAG AAT GCC	2675–2652	NC_012042	[53]
		P—FAM-AAC CCA CAC CAT CCA GGA GCA TCT G-BHQ1	2646–2622	145bp	[53]
MNV	MNV-1	F—CCG CCA TGG TCC TGG AGA ATG-3'	3193–3213	Polyprotein	[54]
		R—GCA CAA CGG CAC TAC CAA TCT TG-3'	3330–3308	DQ285629	[54]
		P—FAM-CGT CGT CGC CTC GGT CCT TGT CAA-BHQ1	3227–3250	138bp	[54]
Mengovirus	MC_0_	F—GCG GGT CCT GCC GAA AGT		5’NTR	[55]
		R—GAA GTA ACA TAT AGA CAG ACG CAC AC	/		[55]
		P—FAM-ATC ACA TTA CTG GCC GAA GC-MGB		100bp	[55]

### RT-qPCR with the CFX96^™^ real time PCR detection system

One-step RT-qPCR amplifications were performed on a CFX96^™^ real time PCR detection system from Bio-Rad (Marnes-la-Coquette, France). Reactions were performed in a 15 μL reaction mixture containing 1X of RNA UltraSense^™^ master mix and 0.63 μL of RNA Ultrasense^™^ enzyme mix, which are components of the RNA UltraSense^™^ One-Step Quantitative RT-PCR System (Life technologies), 2 U RNAse inhibitor (Life technologies), 5 μg of bovine serum albumin (Life Technologies), 500 nM of forward primer, 900 nM of reverse primer, 250 nM of probe, and 5 μL of RNA extract. A negative control containing all the reagents except the RNA template was included with each set of reaction mixtures.

The one-step RT-qPCR program involved 60 min reverse transcription of RNA at 55°C, followed by a 15 min denaturation step at 95°C, 45 cycles of 15 s at 95°C, 1 min at 60°C and 1 min at 65°C. Fluorescence was recorded by the apparatus at the end of the elongation steps (1 minute at 65°C) for each amplification cycle. All samples were characterised by a corresponding Ct value. Negative samples gave no Ct value. A standard curve for each target was generated with synthesized RNA (HAV, HEV, NoV GI, NoV GII, NoV GIV, sapovirus, cosavirus, Aichi virus, human coronavirus (HKU1, 229E, NL63, OC43), TBEV), RNA extracts (rotavirus, astrovirus, enterovirus, MNV and mengovirus), synthesized DNA (parvovirus, bocavirus) or DNA extract (adenovirus) resulting from serial dilution in ultrapure water. The slopes (*S*) of the regression lines were used to calculate the amplification efficiency (*E*) of the RT-qPCR reactions, according to the formula *E* = 10^|-1/s|^-1, to determine the RT-qPCR assay performance.

### RT-dPCR with the BioMark System

Digital PCR works by partitioning a single sample into hundreds of individual PCR reactions. RT-dPCR amplifications were performed on a Fluidigm BioMark System by using qdPCR 37k IFC digital array microfluidic chips (Les Ulis, France). Utilizing nanoscale valves and channels, the Biomark Integrated Fluidic Circuit (IFC) controller partitions each of the 48 samples premixed with PCR reagents into a panel of 770 PCR reaction chambers (i.e. 36,960 individual qPCR reactions on a digital array). By counting the number of positive reactions, the number of target molecules in each sample can be accurately estimated based on the Poisson distribution.

Reactions were performed in a 10 μL reaction mixture containing 1X of RNA UltraSense^™^ master mix, 1X of ROX reference dye and 0.44 μL of RNA Ultrasense^™^ enzyme mix, which are components of the RNA UltraSense^™^ One-Step Quantitative RT-PCR System (Life Technologies), 1X of 20X GE Sample Loading Reagent (Fluidigm), 2 U RNAse inhibitor (Life Technologies), 500 nM of forward primer, 900 nM of reverse primer, 250 nM of probe, and 5.8 μL of RNA extract. A negative control containing all the reagents except the RNA template was included with each set of reaction mixtures. 6 μL out of ten reaction mix was charged onto the chip with the IFC controller MX, but 0.65 μL were effectively partitioned into the 770 chambers of one panel, including 0.38 μL of RNA extract.

The one-step RT-dPCR program involved 60 min reverse transcription of RNA at 55°C, followed by a 15 min denaturation step at 95°C, and lastly 45 cycles of 15 s at 95°C, 1 min at 60°C and 1 min at 65°C. Fluorescence was recorded by the apparatus at the end of the elongation steps (1 minute at 65°C) for each amplification cycle.

The Digital PCR Analysis software (Fluidigm) was used to count the number of positive chambers out of the total number of chambers per panel.

The Poisson distribution was used to estimate the average number of template copies per chamber in a panel [24, 25]. All samples were characterised by a corresponding absolute quantity. No positive chambers were observed in negative samples.

### RT-qPCR with the BioMark System

The 48.48 dynamic arrays were automatically loaded using an integrated fluidic circuit (IFC) controller (Fluidigm Corporation), and real-time reactions were performed and analyzed using a BioMark real-time PCR system and analysis software (Fluidigm Corporation), respectively. As a quality control, negative control samples were included on every array for each viral genome.

RT reactions were performed in a 25 μL reaction mixture containing 1X of First-Strand Buffer, 10mM of DTT and 1 μL of SuperScript^®^ III RT enzyme, which are components of SuperScript^®^ III Reverse Transcriptase (Life technologies), 2 U RNAse inhibitor (Life technologies), 2μM of Random hexamer (Life technologies), 200 μM of dNTP (Life technologies), and 10 μL of nucleic acids. A negative control containing all the reagents except the RNA template was included with each set of reaction mixtures. The RT program involved 5 min at 25°C, followed by 60 min at 55°C, and lastly 15 min at 70°C. Aliquots were kept frozen at -80°C for later use.

Preamplification reactions were performed in a 10 μL reaction mixture containing 1X of SuperMix, a reagent of Perfecta Preamp SuperMix (Quanta), 0.2μl of 0.2X primer pool (1X = 500nM Forward and 900nM Reverse), and 5 μL of cDNA. A negative control containing all the reagents except the cDNA template was included with each set of reaction mixtures. The preamplification program involved 10 min at 95°C, followed by 14 cycles of 15 s at 95°C and 4 min at 6°C. Immediately after the pre-amplification PCR, products were diluted (1:4) and stored at -80°C prior to use in qPCR.

For the qPCR array, 48 x 6 μL reaction mixture containing 1X of RNA UltraSense^™^ master mix, 1X of ROX reference dye and 0.27 μL of RNA UltraSense^™^ enzyme mix, which are components of the RNA UltraSense^™^ One-Step Quantitative RT-PCR System (Life Technologies), 1X of 20X GE Sample Loading Reagent (Fluidigm) and 2.7 μL of DNA extract were charged on the right part of the “48.48 Dynamic Array IFC” plate (BioMark). Negative controls containing all the reagents except the DNA template were included with each set of reaction mixtures. In addition, 48 x 5 μl of a mix of 500 nM of forward primer, 900 nM of reverse primer and 250 nM of probe were deposited on the left part of the plate.

Nine nl of reaction volume mix were charged onto each of the 2304 chambers on the chip with the IFC controller MX.

The qPCR program involved a 15 min denaturation step at 95°C followed by 45 cycles of 15 s at 95°C, 1 min at 60°C and 1 min at 65°C. Fluorescence was recorded by the apparatus at the end of the elongation steps (1 minute at 65°C) for each amplification cycle. Negative samples gave no Ct value.

## Results

### Conventional RT-qPCR and nanofluidic PCR (RT-dPCR, RT-qPCR array)

The sensitivity of conventional qPCR assays targeting 21 viral genomes was compared to the quantitative digital RT-PCR array and to the qualitative nanofluidic real-time PCR array performed on Fluidigm’s BioMark System.

### Quantitative detection by conventional and digital real time RT-PCR assays

Digital RT-PCR’s potential for sensitive and accurate quantification was assessed on 10-fold dilution series of 21 viral genomes (Table 3). The sensitivity was slightly better with RT-qPCR than with RT-dPCR for ten viruses by a factor ranging from 0.3 to 0.9 log_10_ and for four viruses by a factor ranging from 1.3 to 1.6 log_10_. Conversely, sensitivity was better with RT-dPCR than with RT-qPCR for seven viruses by a factor ranging from 0.1 to 1.4 log_10_.

**Table 3:** Comparison of RT-qPCR, RT-dPCR and RT-PCR array assays. Characteristics of standard curves based on the RT-qPCR assays and limit of detection (LOD) of viral targets by RT-qPCR, by RT-dPCR and RT-PCR array assays. The differences between relative quantification (by RT-qPCR) and absolute quantification (by RT-dPCR) were indicated.

Virus	Sample		RT-qPCR (CFX96)					RT-dPCR (FLUIDIGM)			RT-PCR Array (FLUIDIGM)	
	Genome	Type of sample	Slope	E	R^2^	Range of detection (Ct value)	LOD ^a^	LOD ^a^	log_10_(qPCR)-log_10_(dPCR)	Difference between quantification type ^b^	LOD ^a^	log_10_(qPCR)-log_10_(PCR array)
Bocavirus	DNA ss	Plasmidic DNA	-3.20 ± 0.20	106%	0.966	16.90–37.36	0.20	0.80	-0.60	0.17	1.00	-0.70
Parvovirus B19	DNA ss	Plasmidic DNA	-3.05 ± 0.14	113%	0.980	15.22–36.35	0.10	4.00	-1.60	-0.31	1.00	-1.00
Sapovirus	RNA+ ss	Transcript RNA	-3.55 ± 0.29	91%	0.963	20.21–36.01	1.00E+01	4.00E+01	-0.60	0.89	1.00E+02	-1.00
Hepatitis E virus	RNA+ ss	Transcript RNA	-3.49 ± 0.23	93%	0.972	19.91–38.15	1.00E+01	4.00E+01	-0.60	1.25	1.00E+02	-1.00
TBEV	RNA+ ss	Transcript RNA	-3.10 ± 0.21	110%	0.970	21.66–37.54	1.00E+01	7.90E+01	-0.90	1.03	1.00E+04	-3.00
Aichi virus	RNA+ ss	Transcript RNA	-3.34 ± 0.46	99%	0.933	27.18–36.53	1.00E+03	7.89E+02	0.10	1.32	1.00E+05	-2.00
229E	RNA+ ss	Transcript RNA	-3.05 ± 0.37	113%	0.934	25.97–36.89	2.00E+01	3.95E+02	-1.30	1.64	1.00E+03	-1.70
HKU1	RNA+ ss	Transcript RNA	-3.41 ± 0.22	97%	0.982	25.84–36.34	2.00E+01	7.90E+01	-0.60	1.57	1.00E+04	-2.70
Cosavirus	RNA+ ss	Transcript RNA	-3.16 ± 0.27	107%	0.978	24.26–31.41	1.00E+03	7.90E+01	1.10	1.35	1.00E+04	-1.00
Norovirus GGI	RNA+ ss	Transcript RNA	-3.37 ± 0.28	98%	0.973	26.97–38.43	1.00E+02	7.90E+01	0.10	1.44	1.00E+03	-1.00
Norovirus GGIV	RNA+ ss	Transcript RNA	-3.53 ± 0.35	92%	0.952	22.7–37.92	2.00E+03	7.90E+01	1.40	1.51	1.00E+03	0.30
Norovirus GGII	RNA+ ss	Transcript RNA	-3.44 ± 0.32	95%	0.954	23.01–38.53	1.00E+02	7.90E+01	0.10	1.64	1.00E+03	-1.00
OC43	RNA+ ss	Transcript RNA	-3.39 ± 0.31	97%	0.952	22.84–37.70	1.00E+01	3.95E+02	-1.60	1.67	1.00E+04	-3.00
NL63	RNA+ ss	Transcript RNA	-3.31 ± 0.83	100%	0.851	28.59–36.63	2.00E+02	3.95E+02	-0.30	1.97	1.00E+04	-1.70
Hepatitis A virus	RNA+ ss	Transcript RNA	-3.27 ± 0.46	102%	0.916	26.03–39.80	1.00E+02	3.95E+02	-0.60	2.14	1.00E+03	-1.00
Adenovirus 41	DNA ds	Stool	-3.00 ± 0.16	115%	0.986	23.43–35.08	1.00E+02	7.90E+01	0.10	1.47	1.00E+02	0.00
Astrovirus	RNA+ ss	Stool	-3.65 ± 0.54	88%	0.943	32.88–42.23	2.00E+02	7.89E+03	-1.60	3.40	1.00E+05	-2.70
Rotavirus Wa	RNA+ ds	Cell production	-3.51 ± 0.79	93%	0.812	22.05–36.45	1.00E+02	7.89E+02	-0.90	1.56	1.00E+04	-2.00
MNV-1	RNA+ ss	Cell production	-3.01 ± 0.32	115%	0.947	28.33–38.14	1.00E+02	3.95E+02	-0.60	1.95	1.00E+04	-2.00
Enterovirus	RNA+ ss	Cell production	-3.36 ± 0.59	98%	0.894	25.28–36.79	1.00E+03	3.95E+02	0.40	1.97	1.00E+03	0.00
Mengovirus	RNA+ ss	Cell production	-3.21 ± 0.20	105%	0.983	26.77–38.00	1.00E+02	7.89E+02	-0.90	2.05	1.00E+04	-2.00

^a^ LOD expressed as number of genome copies/μl of nucleic acids.

^b^ formula = [log_10_(OD)-log_10_(digital)].

The expected numbers of genome copies calculated *via* the standard curve by RT-qPCR were close to the direct measurement of the target concentrations by RT-dPCR only by testing DNA from plasmids. By testing RNA transcripts, the numbers of genome copies as determined by direct RT-dPCR measurement of the target concentrations were 0.9 to 2.1 log_10_ lower than the expected copy numbers calculated *via* the standard curve by RT-qPCR. Similarly, by testing genomes from viruses in stools and RNA from virus production in cells, the limit of detection (LOD) as determined by RT-dPCR was respectively 1.5 to 3.4 log_10_ and 1.6 to 2.1 log_10_ lower than the expected copy numbers calculated *via* the standard curve by RT-qPCR.

### Sensitive and accurate detection by RT-qPCR array

The potential of the RT-PCR array for sensitive detection was assessed on a dilution series of 21 viral genomes (Table 3). The limits of detection obtained with RT-qPCR array assays ranged from 1 to 10^3^ genome copies / μl of RNA / DNA extracts for 11 viruses and from 10^4^ to 10^5^ genome copies / μl of RNA extracts for the others. RT-qPCR array assays commonly showed a slightly lower sensitivity than conventional RT-qPCR. The sensitivity of both RT-qPCR and RT-qPCR array assays was found to be similar for two viruses (enterovirus, adenovirus 41), and was slightly better with the RT-qPCR than with the RT-qPCR array for 18 viruses by a factor ranging from 0.7 to 3.0 log_10_. Conversely, sensitivity was only 0.3 log_10_ higher with the RT-qPCR array than with conventional RT-qPCR assays for norovirus GIV detection.

### Viral screening by RT-qPCR array and quantitative detection of clinical samples by RT-dPCR

The nanofluid-based (RT)-PCR assays developed were applied to characterize 25 samples (4 culture supernatants and 21 clinical samples previously characterized by NRC) for detection of hepatitis (HAV, HEV) and enteric virus genomes. First, the samples were tested on the RT-PCR array to perform a qualitative screening of the 19 viral genomes. Then the viral-positive samples were specifically quantified by RT-dPCR and by conventional RT-qPCR. Results are shown on Table 4.

**Table 4:** Screening and viral quantification in clinical samples (stools and viral supernatants from cell culture) by RT-qPCR and novel nanofuidic approaches (RT-qPCR and RT-dPCR). Samples were firstly screened by RT-PCR array and then quantified by RT-dPCR. Absolute viral quantification (by RT-dPCR) was compared to relative quantification (by RT-qPCR).

Sample number	Nature	Origine	NRC Virus identification	PCR Array 48x48 detection	RT-dPCR quantification ^a^	RT-qPCR quantification ^a^	Difference between quantification type
HM175/18f	Cell production	ATCC	HAV	3/3	1.51E+09	4.50E+10	1.47
EchoV	Cell production	Echovirus 19. Burke strain	EV	2/2	1.61E+09	1.75E+10	1.04
Adenovirus 40	Cell production	ATCC VR-931	Adenovirus	2/2	7.11E+08	1.42E+10	1.30
Astrovirus GI	Cell production	N/A	Astrovirus	2/2	1.76E+10	2.43E+10	0.14
780627147	stool	HAV/HEV NRC	HAV IA	3/3	1.29E+06	2.30E+06	0.25
1181216151	stool	HAV/HEV NRC	HAV IA	3/3	2.45E+09	7.75E+10	1.50
1280210015	stool	HAV/HEV NRC	HAV IB	3/3	7.52E+07	1.50E+09	1.30
1280514230	stool	HAV/HEV NRC	HAV IB	3/3 (HAV)	2.88E+08	6.85E+09	1.38
				2/2 (Aichi virus)	9.17E+07	2.77E+09	1.48
1280511146	stool	HAV/HEV NRC	HEV 3c	2/2	7.36E+07	1.41E+09	1.28
1280418084	stool	HAV/HEV NRC	HEV 3f	2/2	1.43E+08	1.95E+09	1.13
1280530128	stool	HAV/HEV NRC	HEV 3f	2/2	1.32E+07	5.21E+08	1.60
1280615097	stool	HAV/HEV NRC	HEV 4	2/2	4.44E+07	2.40E+08	0.73
1280522166	stool	HAV/HEV NRC	HEV 3	2/2	2.60E+07	5.34E+09	2.31
E7622	stool	Enteric viruses NRC	RV G12P8	2/2	4.84E+09	7.28E+11	2.18
E8097	stool	Enteric viruses NRC	RV G1P8	2/2	7.83E+08	4.31E+12	3.74
				1/2 (Aichi virus)	7.83E+05	6.00E+07	1.88
E6841	stool	Enteric viruses NRC	Aichi virus	2/2 (Adenovirus)	4.69E+09	5.85E+10	1.10
				2/2 (Astrovirus)	4.42E+08	1.65E+11	2.57
E5486	stool	Enteric viruses NRC	NoV GI.4	3/3	1.98E+07	7.79E+07	0.59
E5569	stool	Enteric viruses NRC	NoV GI.1	3/3	9.58E+06	9.91E+07	1.01
E8050	stool	Enteric viruses NRC	NoV GI.3	3/3	3.96E+07	7.40E+08	1.27
E6929	stool	Enteric viruses NRC	NoV GII.4	3/3	5.53E+07	7.00E+08	1.10
E6618	stool	Enteric viruses NRC	NoV GII.7	3/3	8.29E+05	8.03E+06	0.99
E7859	stool	Enteric viruses NRC	NoV GII.6	3/3	6.94E+07	5.24E+08	0.88
E7022	stool	Enteric viruses NRC	NoV GII.3	3/3	1.95E+07	2.91E+08	1.17
E6992	stool	Enteric viruses NRC	NoV GII.1	3/3	1.75E+06	1.52E+07	0.94
-	stool	Enteric viruses NRC	NoV GII.13	3/3 (NoV GII)	2.95E+06	1.16E+06	-0.41
			+ NoV GIV	2/2 (NoV GIV)	3.04E+05	1.84E+07	1.78

^a^ expressed as number of genome copies/g (stools) or genome copies/mL (cell production).

RT-qPCR array assays detected the previously determined viruses in 100% of the samples. Furthermore, positivity for more than one virus was found in two clinical samples. A stool previously identified as positive for HAV IB was found positive for HAV and Aichi virus and a stool identified as positive for Aichi virus was found positive for Aichi virus, adenovirus and astrovirus. The stool previously identified as co-infected by NoV GII.13 and NoV GIV was confirmed positive for both viruses.

Following the viral screening of 25 samples, the 29 detected viral genomes were successfully quantified by both RT-qPCR and RT-dPCR. The number of genome copies determined for 28 viruses was lower by RT-dPCR with a difference of quantification comprised between 0 and 1 log_10_ for 7 out of the 29 samples (24%), between 1 and 2 log_10_ for 17 out of the 29 samples (59%) and higher than 2 log_10_ for 4 out of the 29 samples (14%). So the numbers of genome copies calculated by absolute quantification (RT-dPCR) were lower than the expected numbers of genome copies calculated by using standard curve of RT-qPCR except in the sample co-infected with NoV GII and NoV GIV. In the latter sample, the NoV GII quantification was 0.4 log_10_ higher by RT-dPCR than by the RT-qPCR assays (1 out of the 29 samples, *i*.*e*. 3%).

## Discussion

Enteric viruses are able to persist for long periods in the environment and can be transmitted with a low infectious dose by human contact, water, food and fomites [26]. They pose a significant public health concern. They are associated with gastroenteritis in humans, but also with hepatitis and other diseases including respiratory infections, conjunctivitis, aseptic meningitis, encephalitis, myocarditis, and paralysis which have high mortality rates, particularly in immunocompromised individuals [27]. Commonly studied groups of enteric viruses include noroviruses and hepatitis viruses, but new tools for detecting the full range of pathogenic viruses are needed for their surveillance in the environment, food samples and for outbreak investigations [28].

Microfluidic digital PCR (RT-dPCR) is an accurate endpoint-sensitive absolute quantification approach that makes it possible to determine the number of target copies without a standard curve. Digital PCR ((RT)-dPCR) was compared to real-time (RT)-PCR for quantifying 19 human enteric viruses and two control process viruses. For detecting viral RNA and cDNA, RT-dPCR assays were often found to be comparable in terms of sensitivity to RT-qPCR.

The number of RNA genome copies determined by digital RT-PCR was often lower than the number of copies expected using spectrophotometry. One potential cause of discrepancy between relative and absolute quantification could be errors introduced by spectrophotometric determination of the nucleic acid concentration, leading to an overestimation of the copy genome number [29, 30]. This could explain why samples from viral stocks and stools potentially containing cellular genomes (non-target RNA) and degraded (non-amplified) targets were particularly affected by quantification discrepancies. Both quantification methods were close when DNA targets were tested. One other potential cause of discrepancy might be the RT step, which is not 100% effective, so that all the RNA may not be transcribed into cDNA and therefore is not quantified by the digital PCR.

Digital RT-PCR may provide more accurate measurements than RT-qPCR, as it is not dependent on amplification efficiency. Moreover, the advantage of this novel technology is that it is more tolerant to inhibitory substances and may reduce the difficulty of quantifying viruses when inhibitors linked to the matrix-type components analysed in food or environmental virology are present [31, 32, 33].

Recent innovations in PCR miniaturization made it possible to conduct high-throughput qPCR in which the reactional volumes are reduced to a nanolitre, leading to a decrease in the cost per assay per sample. Recently, a microfluidic quantitative PCR (MFQPCR) system was developed to simultaneously quantify 11 major human viral pathogens and two process controls (murine norovirus, mengovirus). This system included a specific target amplification (STA) reaction to increase the amount of target genes prior to MFQPCR [34]. In this study, the RT-qPCR array assays were developed and enabled simultaneous detection of 48 samples with 22 targeted virus assays. The preamplification step was also necessary because low amounts of target molecules had to be detected in very small volumes of reaction (9nl). Thus, RT-qPCR array assays involve three separate steps (RT, preamplification and PCR).

Nineteen enteric viruses and two control process viruses (MNV and mengovirus) were targeted. The sensitivity of the RT-qPCR array assays was lower (by 0.8 to 3.8 log_10_) than the limits of detection obtained with conventional RT-qPCR and RT-dPCR. However, all the clinical samples tested with the RT-qPCR array assays were identified and matched the NRC results. Moreover, two stools contained more than one viral genome, and these results completed the NRC analysis. This assay is therefore useful for rapid sample screening.

In conclusion, a combination of RT-qPCR array and RT-dPCR assays could be applied to screen contaminated samples and quantify pathogenic viruses in case of outbreaks investigation and surveillance. The choice of techniques should take into account the aim of analysis, the number of targets involved and the analytical costs. To date, the RT-qPCR array includes enteric viruses frequently reported as the causes of foodborne outbreaks and some additional viruses of lesser epidemiologic importance. In future, this technology could be updated by extending the range of viral targets to gain information during epidemiological studies. For this purpose, BioMark real-time PCR system (Fluidigm) can be also used for high-throughput microfluidic real-time PCR amplification with 96.96 dynamic arrays (Fluidigm) leading to an increase of detected targets. Concerning RT-dPCR assays, it could be helpful for standardizing the quantification of enteric viruses in samples and might be extended to the quantification of other human microbiological pathogens in foods.

## References

[1] Kotwal G, Cannon JL. Environmental persistence and transfer of enteric viruses. Curr Opin Virol. 2014; 4C: 37–43. 10.1016/j.coviro.2013.12.003 24413147

[2] Gibson KE. Viral pathogens in water: occurrence, public health impact, and available control strategies. Curr Opin Virol. 2014; 4: 50–57. 10.1016/j.coviro.2013.12.005 24440908 PMC7185559

[3] Koo HL, Ajami N, Atmar RL, DuPont HL. Noroviruses: The leading cause of gastroenteritis worldwide. Discov Med. 2010; 10: 61–70. 20670600 PMC3150746

[4] Matthews JE, Dickey BW, Miller RD, Felzer JR, Dawson BP, Lee AS, et al The epidemiology of published norovirus outbreaks: a review of risk factors associated with attack rate and genogroup. Epidemiol Infect. 2012; 140: 1161–1172. 10.1017/S0950268812000234 22444943 PMC3350621

[5] Nainan OV, Xia G, Vaughan G, Margolis HS. Diagnosis of hepatitis a virus infection: a molecular approach. Clin Microbiol Rev. 2006; 19: 63–79. 16418523 10.1128/CMR.19.1.63-79.2006 PMC1360271

[6] Van der Poel WHM. Food and environmental routes of Hepatitis E virus transmission. Curr Opin Virol. 2014; 4: 91–96. 10.1016/j.coviro.2014.01.006 24513966

[7] Vaughan G, Goncalves Rossi LM, Forbi JC, de Paula VS, Purdy MA, Xia G, et al Hepatitis A virus: Host interactions, molecular epidemiology and evolution. Infect Genet Evol. 2014; 21C: 227–243. 10.1016/j.meegid.2013.10.023 24200587

[8] Bibby K, Peccia J. Identification of viral pathogen diversity in sewage sludge by metagenome analysis. Environ Sci Technol. 2013; 47: 1945–1951. 10.1021/es305181x 23346855 PMC3963146

[9] Lindquist L. Tick-borne encephalitis. Handb Clin Neurol. 2014; 123: 531–559. 10.1016/B978-0-444-53488-0.00025-0 25015503

[10] Malik YS, Kumar N, Sharma K, Dhama K, Shabbir MZ, Ganesh B, et al Epidemiology, phylogeny, and evolution of emerging enteric Picobirnaviruses of animal origin and their relationship to human strains. BioMed Res Int. 2014; 2014: 780752 10.1155/2014/780752 25136620 PMC4124650

[11] Oka T, Wang Q, Katayama K, Saif LJ. Comprehensive Review of Human Sapoviruses. Clin Microbiol Rev. 2015; 28: 32–53. 10.1128/CMR.00011-14 25567221 PMC4284302

[12] Atmar RL, Estes MK. Diagnosis of noncultivatable gastroenteritis viruses, the human caliciviruses. Clin Microbiol Rev. 2001; 14: 15–37. 11148001 10.1128/CMR.14.1.15-37.2001 PMC88960

[13] Sánchez G, Bosch A, Pintó RM. Hepatitis A virus detection in food: current and future prospects. Lett Appl Microbiol. 2007; 45: 1–5. 17594452 10.1111/j.1472-765X.2007.02140.x

[14] Lees D, CEN WG6 TAG4. International standardization of a method for detection of human pathogenic viruses in molluscan shellfish. Food Environ Virol. 2010; 2: 146–155.

[15] ISO/TS 15216–1. Microbiology of food and animal feed—Horizontal method for determination of hepatitis A virus and norovirus in food using real-time RT-PCR—Part 1: Method for quantification. International Organization for Standardization, Geneva, Switzerland. 2013.

[16] ISO/TS 15216–2. Microbiology of food and animal feed—Horizontal method for determination of hepatitis A virus and norovirus in food using real-time RT-PCR—Part 2: Method for qualitative detection. International Organization for Standardization, Geneva, Switzerland. 2013.

[17] Lemon SM, Murphy PC, Shields PA, Ping LH, Feinstone SM, Cromeans T, et al Antigenic and genetic variation in cytopathic hepatitis A virus variants arising during persistent infection: evidence for genetic recombination. J Virol. 1991; 65: 2056–2065. 1705995 10.1128/jvi.65.4.2056-2065.1991 PMC240056

[18] Cromeans T, Sobsey MD, Fields HA. Development of a plaque assay for a cytopathic, rapidly replicating isolate of hepatitis A virus. J Med Virol. 1987; 22: 45–56. 3035079 10.1002/jmv.1890220107

[19] Dubois E, Hennechart C, Deboosère N, Merle G, Legeay O, Burger C, et al Intra-laboratory validation of a concentration method adapted for the enumeration of infectious F-specific RNA coliphage, enterovirus, and hepatitis A virus from inoculated leaves of salad vegetables. Int J Food Microbiol. 2006; 108: 164–171. 16387377 10.1016/j.ijfoodmicro.2005.11.007

[20] Cannon JL, Papafragkou E, Park GW, Osborne J, Jaykus LA, Vinjé J. Surrogates for the study of norovirus stability and inactivation in the environment: a comparison of murine norovirus and feline calicivirus. J Food Prot. 2006; 69: 2761–2765. 17133824 10.4315/0362-028x-69.11.2761

[21] Wobus CE, Karst SM, Thackray LB, Chang KO, Sosnovtsev SV, Belliot G, et al Replication of Norovirus in cell culture reveals a tropism for dendritic cells and macrophages. PLoS Biol. 2004; 2: e432 15562321 10.1371/journal.pbio.0020432 PMC532393

[22] Costafreda MI, Bosch A, Pinto RM. Development, evaluation and standardization of a real time TaqMan reverse transcription-PCR assay for quantification of hepatitis A virus in clinical and shellfish samples. App Environ Microbiol. 2006; 72: 3846–3855. 10.1128/AEM.02660-05 PMC1489592 16751488

[23] Coudray-Meunier C, Fraisse A, Martin-Latil S, Guillier L, Perelle S. Discrimination of infectious hepatitis A virus and rotavirus by combining dyes and surfactants with RT-qPCR. BMC Microbiol. 2013; 13: 216 10.1186/1471-2180-13-216 24083486 PMC3853579

[24] Dube S, Qin J, Ramakrishnan R. Mathematical analysis of copy number variation in a DNA sample using digital PCR on a nanofluidic device. PLoS One. 2008; 3(8):e2876 10.1371/journal.pone.0002876 18682853 PMC2483940

[25] Bhat S, Herrmann J, Armishaw P, Corbisier P, Emslie KR. Single molecule detection in nanofluidic digital array enables accurate measurement of DNA copy number. Anal Bioanal Chem. 2009; 394: 457–467. 10.1007/s00216-009-2729-5 19288230

[26] Koopmans M, Duizer E. Foodborne viruses: an emerging problem. Int J Food Microbiol. 2004; 90: 23–41. 14672828 10.1016/S0168-1605(03)00169-7 PMC7127053

[27] Griffin DW, Donaldson KA, Paul JH, Rose JB. Pathogenic human viruses in coastal waters. Clin Microbiol Rev. 2003; 16: 129–143. 12525429 10.1128/CMR.16.1.129-143.2003 PMC145303

[28] Kocwa-Haluch R. Waterborne enteroviruses as a hazard for human health. PJoES. 2001; 10: 485–487.

[29] Henrich TJ, Gallien S, Li JZ, Pereyra F, Kuritzkes DR. Low-level detection and quantitation of cellular HIV-1 DNA and 2-LTR circles using droplet digital PCR. J Virol Methods. 2012; 186: 68–72. 10.1016/j.jviromet.2012.08.019 22974526 PMC3517891

[30] Sanders R, Mason DJ, Foy CA, Huggett JF. Evaluation of digital PCR for absolute RNA quantification. PLoS One. 2013; 8: e75296 10.1371/journal.pone.0075296 24073259 PMC3779174

[31] Coudray-Meunier C, Fraisse A, Martin-Latil S, Guillier L, Delannoy S, Fach P, et al A comparative study of digital RT-PCR and RT-qPCR for quantification of Hepatitis A virus and Norovirus in lettuce and water samples. Int J Food Microbiol. 2015; 201:17–26. 10.1016/j.ijfoodmicro.2015.02.006 25725459

[32] Rački N, Morisset D, Gutierrez-Aguirre I, Ravnikar M. One-step RT-droplet digital PCR: a breakthrough in the quantification of waterborne RNA viruses. Anal Bioanal Chem. 2014; 406: 661–667. 10.1007/s00216-013-7476-y 24276251 PMC3892107

[33] Morisset D, Štebih D, Milavec M, Gruden K, Žel J. Quantitative analysis of food and feed samples with droplet digital PCR. PLoS One. 2013; 8: e62583 10.1371/journal.pone.0062583 23658750 PMC3642186

[34] Ishii S, Kitamura G, Segawa T, Kobayashi A, Miura T, Sano D, et al Microfluidic quantitative PCR for simultaneous quantification of multiple viruses in environmental water samples. Appl Environ Microbiol. 2014; 80: 7505–7511. 10.1128/AEM.02578-14 25261510 PMC4249240

[35] Martin-Latil S, Hennechart Collette C, Guillier L, Perelle S. Duplex RT-qPCR for the detection of hepatitis E virus in water, using a process control. Int J Food Microbiol. 2012; 157: 167–73. 10.1016/j.ijfoodmicro.2012.05.001 22633799

[36] Pang XL, Cao M, Zhang M, Lee B. Increased sensitivity for various rotavirus genotypes in stool specimens by amending three mismatched nucleotides in the forward primer of a real-time RT-PCR assay. J Virol Methods. 2010; 172: 85–87. 10.1016/j.jviromet.2010.12.013 21185331

[37] Pang XL, Lee B, Boroumand N, Leblanc B, Preiksaitis JK, Yu Ip CC. Increased detection of rotavirus using a real time reverse transcription-polymerase chain reaction (RT-PCR) assay in stool specimens from children with diarrhea. J Med Virol. 2004; 72: 496–501. 14748075 10.1002/jmv.20009

[38] da Silva AK, Le Saux JC, Parnaudeau S, Pommepuy M, Elimelech M, Le Guyader FS. Evaluation of removal of noroviruses during wastewater treatment, using real-time reverse transcription-PCR: different behaviors of genogroups I and II. Appl Environ Microbiol. 2007; 73: 7891–7897. 17933913 10.1128/AEM.01428-07 PMC2168159

[39] Svraka S, Duizer E, Vennema H, de Bruin E, van der Veer B, Dorresteijn B, et al Etiological role of viruses in outbreaks of acute gastroenteritis in The Netherlands from 1994 through 2005. J Clin Microbiol. 2007; 45: 1389–1394. 17360839 10.1128/JCM.02305-06 PMC1865895

[40] Loisy F, Atmar RL, Guillon P, Le Cann P, Pommepuy M, Le Guyader FS. Real-time RT-PCR for norovirus screening in shellfish. J Virol Methods. 2005; 123: 1–7. 15582692 10.1016/j.jviromet.2004.08.023

[41] Kageyama T, Kojima S, Shinohara M, Uchida K, Fukushi S, Hoshino FB, et al Broadly reactive and highly sensitive assay for Norwalk-like viruses based on real-time quantitative reverse transcription-PCR. J Clin Microbiol. 2003; 41: 1548–1557. 12682144 10.1128/JCM.41.4.1548-1557.2003 PMC153860

[42] Trujillo AA, McCaustland KA, Zheng DP, Hadley LA, Vaughn G, Adams SM, et al Use of TaqMan real-Time Reverse transcription-PCR for rapid detection, quantification, and typing of norovirus. J Clin Microbiol. 2006; 44: 1405–1412. 16597869 10.1128/JCM.44.4.1405-1412.2006 PMC1448641

[43] Chan MC, Sung JJ, Lam RK, Chan PK, Lai RW, Leung WK. Sapovirus detection by quantitative real-time RT-PCR in clinical stool specimens. J Virol Methods. 2006; 134:146–153. 16427707 10.1016/j.jviromet.2005.12.013

[44] Drexler J, Baumgarte S, de Souza Luna LK, Eschbach-Bludau M, Lukashev AN, Drosten C. Aichi Virus Shedding in High Concentrations in Patients with Acute Diarrhea. Emerg Infect Dis. 2011; 17: 1544–1548. 10.3201/eid1708.101556 21801647 PMC3381558

[45] van Maarseveen NM, Wessels E, de Brouwer CS, Vossen AC, Claas EC. Diagnosis of viral gastroenteritis by simultaneous detection of Adenovirus group F, Astrovirus, Rotavirus group A, Norovirus genogroups I and II, and Sapovirus in two internally controlled multiplex real-time PCR assays. J Clin Virol. 2010; 49: 205–210. 10.1016/j.jcv.2010.07.019 20829103

[46] Gaunt ER, Hardie A, Claas EC, Simmonds P, Templeton KE. Epidemiology and clinical presentations of the four human coronaviruses 229E, HKU1, NL63, and OC43 detected over 3 years using a novel multiplex real-time PCR method. J Clin Microbiol. 2010; 48: 2940–2947. 10.1128/JCM.00636-10 20554810 PMC2916580

[47] Zlateva KT, Crusio KM, Leontovich AM, Lauber C, Claas E, Kravchenko AA, et al Design and validation of consensus-degenerate hybrid oligonucleotide primers for broad and sensitive detection of corona- and toroviruses. J Virol Methods. 2011; 77: 174–83. 10.1016/j.jviromet.2011.08.005 PMC7112876 21864579

[48] Schwaiger M, Cassinotti P. Development of a quantitative real-time RT-PCR assay with internal control for the laboratory detection of tick borne encephalitis virus (TBEV) RNA. J Clin Virol. 2003; 27: 136–145. 12829035 10.1016/s1386-6532(02)00168-3

[49] Kaida A, Kubo H, Sekiguchi J, Ohyama M, Goto K, Hase A, et al Detection of Five Rash-Associated Viruses Using Multiplex Real-Time PCR during 2006–2011. Jpn J Infect Dis. 2012; 65: 430–432. 22996218

[50] Mohamed N, Elfaitouri A, Fohlman J, Friman G, Blomberg J. A sensitive and quantitative single-tube real-time reverse transcriptase-PCR for detection of enteroviral RNA. J Clin Virol. 2004; 30: 150–156. 15125871 10.1016/j.jcv.2003.08.016

[51] Monpoeho S, Dehee A, Mignotte B, Schwartzbrod L, Marechal V, Nicolas JC, et al Quantification of enterovirus RNA in sludge samples using single tube real-time RT-PCR. Biotechniques. 2000; 29: 88–93. 10907082 10.2144/00291st03

[52] Stöcker A, Souza BF, Ribeiro TC, Netto EM, Araujo LO, Corrêa JI, et al Cosavirus infection in persons with and without gastroenteritis, Brazil. Emerg Infect Dis. 2012; 18: 656–659. 10.3201/eid1804.111415 22469070 PMC3309695

[53] Xu ZQ, Cheng WX, Li BW, Li J, Lan B, Duan ZJ. Development of a Real-Time PCR Assay for Detecting and Quantifying Human Bocavirus 2. J Clin Microbiol 2011; 49: 1537–1541. 10.1128/JCM.00196-10 21325551 PMC3122821

[54] Martin-Latil S, Hennechart Collette C, Guillier L, Perelle S. Comparison of two extraction methods for the detection of hepatitis A virus in semi-dried tomatoes and murine norovirus as a process control by duplex RT-qPCR. Food Microbiol. 2012; 31: 246–253. 22608230 10.1016/j.fm.2012.03.007

[55] Pintó RM, Costafreda MI, Bosch A. Risk assessment in shellfish-borne outbreaks of hepatitis A. J Appl Environ Microbiol. 2009; 75: 7350–7355. 10.1128/AEM.01177-09 PMC2786421 19820160

